# Depression detection using deep learning and large language models from multimodalities

**DOI:** 10.3389/fdgth.2026.1759857

**Published:** 2026-03-09

**Authors:** Yasir Hussain, Muhammad Asad Zaheer, Ayaz Muhammad Khan, Aamir Saeed Malik

**Affiliations:** 1 Faculty of Information Technology, Brno University of Technology, Brno, Czech Republic; 2 Division of Education, University of Education, Lahore, Pakistan

**Keywords:** affective computing, deep learning architectures, EEG-based classification, large language models, multimodal depression detection

## Abstract

Depression is a complex psychiatric disorder that affects neural functioning, cognition, emotion, and behavior, making objective assessment a persistent clinical challenge. Traditional diagnostic methods depend on subjective interpretation, whereas recent advances in deep learning have enabled automated, data-driven detection across physiological and behavioral modalities. Among unimodal approaches, electroencephalography (EEG) remains the most widely used due to its sensitivity to depression-related neurophysiological alterations. However, EEG models often rely on small, homogeneous datasets and controlled laboratory conditions, limiting their generalizability. Multimodal architectures that integrate speech, facial expression, and EEG features provide richer representations and consistently outperform single-modality systems. Transformer-based fusion mechanisms and attention-guided models effectively capture complementary cross-modal cues, achieving 90%–95% accuracy on controlled laboratory datasets such as SEED-IV, while yielding more conservative F1-scores of approximately 0.80–0.90 on ecologically valid community datasets such as DAIC-WOZ. The emergence of Large Language Models (LLMs) represents a further methodological shift, offering cross-modal alignment, contextual inference, and data-efficient adaptation through unified embedding spaces and few-shot capabilities. This mini-review synthesizes recent advances in EEG-based, multimodal, and LLM-driven depression detection. It evaluates how modality diversity and architectural sophistication enhance performance while critically examining persisting limitations in dataset diversity, standardization, interpretability, and clinical validation. The convergence of multimodal deep learning with LLM reasoning signals a promising direction toward scalable, explainable, and clinically deployable AI systems for the assessment of objective depression.

## 1 Introduction

Depression is a widespread psychiatric disorder affecting more than 300 million individuals and impairing emotional, cognitive, and functional well-being (1). Its global economic burden exceeds $1 trillion annually, underscoring the need for objective and scalable diagnostic methods (2). Current assessments rely heavily on clinical interviews and self-report instruments, which are vulnerable to cultural, linguistic, and subjective biases (3, 4). The absence of consistent neurophysiological biomarkers further limits early identification and personalized treatment planning.

Recent developments in neurophysiology and behavioral sciences have created new opportunities for data-driven depression assessment. Neuroimaging modalities such as MRI, fMRI, and PET have demonstrated structural and functional abnormalities associated with depressive states (5–7), yet their high cost and limited temporal resolution reduce feasibility in routine clinical settings. Electroencephalography (EEG), in contrast, is inexpensive, noninvasive, and temporally precise, enabling the capture of oscillatory disturbances and network-level dysfunction linked to depression (8).

Behavioral modalities—including speech, facial expressions, text, and handwriting—offer complementary information reflecting prosodic changes, micro-expressions, linguistic patterns, and psychomotor slowing. When combined with neural signals, these behavioral cues improve ecological validity and provide a richer characterization of affective and cognitive symptoms.

Deep learning (DL) has accelerated automated depression detection by enabling hierarchical extraction of spatiotemporal features from raw EEG and behavioral data. Architectures such as CNNs, RNNs, and transformers show strong performance and generalizability compared with traditional handcrafted methods (9–11). Consequently, DL-based EEG analysis has become central to computational mental health research.

Large Language Models (LLMs) represent a further shift by supporting multimodal integration, clinical text interpretation, conversational assessment, and explainable reasoning (12). Their ability to combine EEG-derived features with behavioral and linguistic cues enables more holistic and individualized diagnostic frameworks (13).

Despite progress, key gaps remain concerning modality reliability, multimodal robustness, the comparative value of EEG as a standalone signal, and the capacity of DL and LLM architectures to integrate heterogeneous data streams for real-time clinical use. Addressing these issues is essential for developing scalable and trustworthy mental health assessment tools.

This review synthesizes recent advances in EEG-based, behavioral, and LLM-enhanced approaches to evaluate how modality expansion and emerging learning architectures improve accuracy, interpretability, and clinical relevance. While EEG remains the most physiologically informative unimodal modality, multimodal fusion combined with LLM-enabled contextual reasoning appears most promising for clinically reliable automated depression detection.

### 1.1 Literature search strategy

This mini-review conducted a structured search across PubMed, IEEE Xplore, Scopus, and Google Scholar, focusing on studies published between 2016 and 2025. Keywords included depression detection, deep learning, EEG analysis, multimodal learning, speech-based depression, facial expression analysis, text, handwriting, and large language models.

Priority was given to peer-reviewed studies that used deep learning or multimodal architectures involving behavioral or neurophysiological modalities, such as EEG, speech, facial expressions, text, handwriting, or sensor-derived signals. Traditional machine learning studies were included only when they were helpful in a historical or comparative context. Selection emphasized methodological transparency, dataset diversity, and clinical relevance. Each study was assessed for bias and quality based on sample size, validation method, reproducibility, and data accessibility. A detailed summary appears in Appendix Table A1., outlining key characteristics and bias ratings. Reference lists were manually screened to identify additional relevant work. As this review adopts a narrative and exploratory approach, no quantitative meta-analysis was performed.

## 2 Deep learning strategies

Deep learning provides an end-to-end framework for modeling neurophysiological and behavioral signals associated with depression. By learning hierarchical representations from raw or minimally processed data, DL methods overcome the limitations of handcrafted features and capture complex spatiotemporal patterns essential for automated assessment.

### 2.1 EEG-based deep learning

EEG remains a central modality in DL-based depression research due to its temporal precision, accessibility, and direct measurement of neural oscillations. Convolutional, recurrent, and transformer-based models effectively extract multichannel EEG features (9–11), identifying biomarkers such as altered alpha–beta ratios, disrupted frontotemporal coupling, and hemispheric asymmetry. Although EEG is physiologically informative, speech- and video-based systems trained on datasets such as AVEC and DAIC–WOZ achieve comparable performance (F1 > 0.85) (14, 15), demonstrating the value of behavioral signals.

Hybrid architectures, including CNN–LSTM and attention-guided models (16), enhance temporal representation and cross-subject generalization. Domain adaptation and transfer learning mitigate variability across subjects and recording settings (17, 18). Graph-based methods further model EEG as functional connectivity networks, with graph convolutional and multi-scale GNNs identifying topological disruptions linked to depression and improving interpretability through neurophysiological relevance (18).

Beyond conventional spectral biomarkers, recent studies highlight the importance of non-linear complexity and hemispheric connectivity lateralization as sensitive indicators of depressive neurodynamics. Measures such as multiscale entropy, fractal dimension, and Lempel–Ziv complexity capture irregularity and reduced neural adaptability that may not be visible in standard oscillatory power analysis. In parallel, research on effective connectivity lateralization demonstrates that depression is associated with disrupted inter-hemispheric information flow and asymmetric network efficiency, particularly in fronto-limbic circuits. These complexity- and lateralization-based biomarkers provide complementary physiological insight and are increasingly integrated into graph-based and deep learning frameworks to enhance sensitivity to subtle cognitive and affective alterations.

### 2.2 Multimodal deep learning integration

DL approaches also extend to behavioral modalities such as speech, facial expressions, text, and handwriting. Speech models capture prosodic and spectral indicators of affective alterations (19). Facial-expression analysis using CNNs and LSTMs identifies micro-expressions and reduced emotional expressivity (20). Transformer-based language models extract linguistic markers reflecting cognitive and emotional patterns (19), while handwriting dynamics, including stroke speed and pressure, reveal psychomotor slowing.

Multimodal DL integrates diverse signals to provide more robust, ecologically valid assessments. Cross-modal fusion combines neural, acoustic, visual, linguistic, and motor cues, yielding more stable predictions and consistently outperforming unimodal models, as summarized in Table 1.

**Table 1:** Representative deep learning approaches for EEG-based depression detection.

Study	Modality	Model	Key insights
Acharya et al. (21)	EEG	CNN	Learned spatial-temporal EEG patterns and hemispheric asymmetry in depression.
Mumtaz and Qayyum (22)	EEG	CNN	Automated feature extraction reveals discriminative depressive EEG oscillations.
Kang et al. (23)	EEG	CNN+Explainability	Identified key EEG biomarkers through post-hoc feature attribution.
Ay et al. (16)	EEG	CNN-LSTM	Jointly modeled short-term and long-term temporal EEG dependencies.
Seal et al. (24)	EEG	DeprNet (CNN)	Revealed right-hemisphere dominance; high accuracy on short raw EEG windows.
Li et al. (25)	Handwriting	ML (handwriting feature-based classifier)	Electronic handwriting from 21 psychological tasks (position, pressure, speed, tilt); derived kinematic features to distinguish MDD patients from healthy controls with high screening accuracy.
Kumar and Ghosh (26)	Handwriting	Recurrent neural network (RNN)	Represented handwriting trajectories as functional graphs; identified disruptions in motor coordination and pressure variability linked to depressive states.
Uyulan et al. (27)	EEG	Compact CNN	Accurate severity classification with lightweight architecture.
Rafiei et al. (28)	EEG	InceptionTime	Eyes-closed EEG more stable; 1 min recordings sufficient for detection.
Song et al. (17)	EEG	LSDD-EEGNet	Domain adaptation reduces subject variability using frontal channels.
Xia et al. (18)	EEG	Attention-CNN	Multi-head attention captures inter-channel dependencies and improves interpretability.
Jo and Kwak (29)	Speech+Text	CNN+BiLSTM (Four-stream)	The four-stream model, which fuses acoustic and linguistic embeddings, improves classification performance by 11%.
Zhang et al. (15)	Audio+Video	CNN-based audio-video fusion	DepITCM integrates temporal–spatial visual cues with acoustic features, thereby enhancing the accuracy of depression detection in multimodal settings.
Zhang et al. (20)	Audio+Video+Text	Deep multimodal network	A unified multimodal framework combining audio prosody, facial dynamics, and linguistic content to improve depression risk estimation.
Zhang et al. (14)	Audio+Video+Text	Multi-level attention network	An attention-driven fusion architecture that jointly learns cross-modal correlations across text, speech, and video for depression prediction.

### 2.3 Toward multimodal and LLM-enabled models

DL-based feature extraction naturally expands into systems that combine multimodal input with the reasoning capabilities of LLMs. Unlike earlier architectures that process modalities independently, LLM-driven frameworks support unified embeddings, cross-modal attention, and contextual fusion. These capabilities enhance interpretability, adaptability, and scalability, enabling coherent integration of the EEG.

Across the reviewed studies, EEG consistently appears as the strongest unimodal signal for depression detection, achieving 88%–99% accuracy with deep learning models (21). These results reflect EEG’s ability to capture fast neural dynamics, oscillatory activity, and hemispheric asymmetries linked to depressive states. Although EEG performs well on its own, multimodal approaches show that combining EEG with speech, facial expressions, or text improves stability and generalizability by leveraging complementary behavioral cues. Even when multimodal studies report regression metrics, the trend remains clear: increasing modality diversity strengthens robustness and predictive performance. Multimodal systems based on LLM further enhance this by integrating heterogeneous inputs into a unified representation space for more context-aware inference (15). Thus, while EEG is the most informative unimodal modality, multimodal fusion provides measurable gains and greater resilience to noise, subject variability, and real-world conditions.

## 3 Large language model strategies for depression detection

LLMs have become a powerful extension of deep learning frameworks for detecting depression. Recent research has highlighted the performance advantages of LLMs over traditional deep learning methods in depression detection. For example, a study by (30) demonstrated that fine-tuned GPT models significantly outperformed CNN- and LSTM-based models on the DAIC-WOZ dataset (31), highlighting their superior handling of linguistic and behavioral cues in depression detection. Unlike single-modality architectures, LLMs integrate heterogeneous data sources—EEG, speech, facial expressions, handwriting, and text—into a unified representation space, allowing richer contextual reasoning and scalable multimodal assessment. Through pretrained knowledge and cross-modal alignment, LLMs support interpretable, data-efficient, and clinically meaningful depression evaluation.

LLMs enhance deep learning by providing contextual reasoning, shared embeddings, and cross-modal alignment across EEG patterns, acoustic cues, facial dynamics, textual content, and motor behavior. Their ability to perform few-shot learning, domain adaptation, and explainable prediction makes them particularly suitable for real-world clinical environments with limited labeled data. The integration of speech features, such as acoustic landmarks, into LLMs has been shown to improve the detection of depression (34). This multimodal approach outperformed traditional deep learning models like CNNs and LSTMs, achieving higher F1 scores and better generalization across varied datasets, especially when applied to the DAIC-WOZ dataset.

### 3.1 LLM architectures for multimodal fusion and explainability

LLMs effectively capture linguistic, emotional, and cognitive markers of depression. Models such as BERT (32), RoBERTa, DistilBERT, and GPT (33) variants identify depressive language through cues like self-referential expressions, negative affect, and cognitive distortions. Combined with speech encoders such as Wav2Vec2 (34) or HuBERT, LLMs merge semantic and prosodic signals for richer language-based assessment.

A key strength of LLMs is their ability to fuse heterogeneous modalities using cross-attention, shared embeddings, and multimodal alignment mechanisms. This enables the integration of EEG features, acoustic representations, facial micro-expressions, gaze patterns, handwriting kinematics, textual content, and sensor-derived behavioral data (35). Empirical evidence shows improved accuracy and robustness as more modalities are combined, demonstrating the synergistic value of multimodal LLM-driven frameworks.

**Figure 1:**
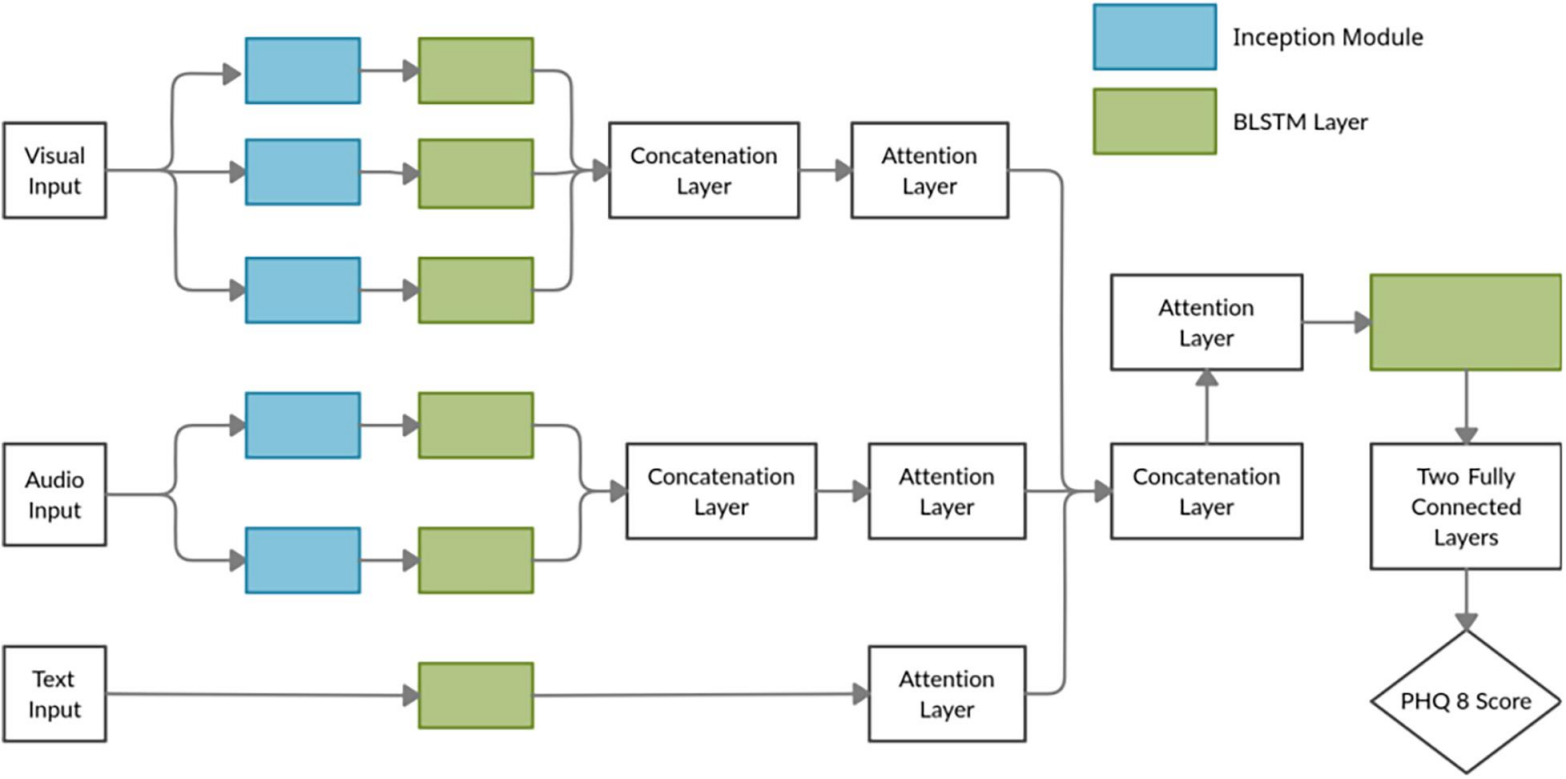
DepressNet: Multimodal hierarchical attention mechanism for visual, audio, and textual fusion. Reproduced with permission from “DepressNet: Multimodal Hierarchical Attention mechanism-based network” by Guramritpal Singh Saggu, Keshav Gupta and K. V. Arya.

Figure 1 illustrates a typical fusion architecture in which modality-specific encoders project audio, visual, and textual inputs into a shared latent space, with attention-based mechanisms enhancing both interpretability and prediction. Evidence from MODMA and E-DAIC indicates that LLM-enabled fusion consistently surpasses unimodal approaches.

LLMs further contribute to explainability and high-level reasoning through zero- and few-shot inference, chain-of-thought generation, and contextual understanding (37). Efficient fine-tuning methods such as LoRA (38), QLoRA (39), and CORAL (40) enable practical clinical deployment, while attention-based outputs highlight salient EEG, speech, and facial features.

Compared with conventional deep learning architectures that operate on fixed feature representations, LLM-based multimodal frameworks demonstrate superior contextual integration and interpretability. For example, LLM-driven systems that jointly process interview transcripts, speech signals, and facial dynamics have shown improved severity prediction and cross-modal consistency relative to traditional fusion pipelines that rely on static embeddings or late-fusion strategies (35) unlike CNN- or LSTM-based models that primarily learn correlation patterns, LLMs leverage pretrained semantic knowledge to align behavioral cues with linguistic and affective context, enabling more clinically interpretable reasoning pathways. This shift from feature-level fusion to reasoning-aware multimodal modeling represents a key methodological advancement in depression detection.

As shown in Table 2, multimodal deep learning models that integrate audio, visual, textual, and behavioral signals consistently outperform unimodal systems, underscoring the benefits of combining complementary modalities for a more robust, holistic evaluation of depression.

**Table 2:** Deep learning strategies for multimodal depression detection across speech, video, text, and behavioral cues.

Study	Modality	Model	Key insights
Sadeghi et al. (35)	Audio+Text+Facial Expressions	Large Language Model	Improves depression severity prediction by combining interview transcripts and facial data.
Li et al. (41)	Speech	Large Language Model+Psychological Knowledge	Enhances depression recognition by integrating audio features with psychological knowledge on the DAIC-WOZ dataset.
Li et al. (42)	Audio+ Video+ Expert Features	Flexible parallel transformer (FPT-Former)	Multimodal transformer with expert priors improves PHQ-8 prediction accuracy and robustness.
Ding et al. (32)	Audio+ Text	BERT-CNN+Audio Transformer	Cross-modal attention fusion of speech and linguistic embeddings achieved F1=0.90 on DAIC-WOZ dataset.
Yin et al. (19)	Speech	Parallel CNN+Transformer	Combines local spectral and temporal features, enhancing speech-based depression detection.
Zhang et al. (43)	Audio+Video	Multimodal Large Language Model (MLlm-DR)	Integrates speech and visual data to provide explainable depression diagnosis, achieving state-of-the-art results on CMDC and E-DAIC-WOZ datasets.
Lu and Koehn (44)	Text+Audio+ Video	LLM2Vec+Multimodal LLM	Combines text, audio, and visual data for robust depression detection.
Park and Moon (45)	Speech+Text	BERT-CNN+CNN-BiLSTM	Attention-based feature fusion improves detection performance and interpretability on DAIC-WOZ.
Lam et al. (46)	Audio+Text	CNN+Transformer	A context-aware deep learning framework that integrates conversational and prosodic features for improved depression detection.
Qin et al. (47)	Text+Interview Data	Large Language Model (LlaMADRS)	Automates depression severity assessment using zero-shot prompting, achieving high agreement with clinician ratings.

While this manuscript primarily discusses general Large Language Models (LLMs) in depression detection, it is essential to highlight recent advancements in Brain-LLM frameworks that have been specifically adapted to work with physiological signals. These specialized models bridge the gap between general-purpose LLMs and the unique requirements of neural decoding tasks. Notable examples include MindLLM (41, 48), EEG Emotion Copilot (48), BrainEC-LLM (49), and EEG-GPT (50), which integrate LLMs with physiological data, particularly EEG signals, to enhance emotional recognition, brain-state classification, and mental health diagnostics.

For instance, MindLLM utilizes EEG data in conjunction with LLMs to enable real-time recognition of emotional states (41), while BP-GPT adapts LLMs for interpreting brain activity patterns related to cognitive and emotional states (48). Similarly, EEG Emotion Copilot uses EEG signals alongside LLMs to accurately detect emotional responses, providing valuable insights into emotional regulation (51). Furthermore, BrainEC-LLM and EEG-GPT showcase how LLMs can be fine-tuned for the interpretation of complex neural data, leading to more personalized and clinically relevant outcomes (49, 50)

These advancements signify a shift from general-purpose LLMs to specialized frameworks that are optimized for the complexities of neural data, marking significant progress in the fields of neuropsychology, mental health assessment, and brain-computer interface (BCI) technologies.

### 3.2 Cross-study comparative synthesis

Table 3 highlights consistent trends across modality choices and model architectures. Overall, differences in reported accuracy are primarily driven by dataset quality, participant diversity, and evaluation protocols rather than the intrinsic superiority of specific modalities. This reinforces the need for standardized benchmarking across studies.

**Table 3:** Cross-study synthesis of modality combinations, model architectures, and performance reliability among representative studies.

Modality type	Typical accuracy/F1	Best architecture	Generalizability	Key studies
EEG (unimodal)	88%–99%	CNN/CNN-LSTM	Moderate (risk of overfitting in small datasets)	Acharya et al. (21); Uyulan et al. (27)
Speech (unimodal)	75%–90%	CNN, Transformer	Good with domain adaptation; sensitive to noise and language variability	Yin et al. (19)
Text (LLM-based)	70%–85%	BERT, RoBERTa	High interpretability and semantic depth; dependent on data quality	Hussain et al. (52); Pilicita and Barra (53)
Multimodal (EEG + Speech/Text)	90%–95%	Transformer/Attention Network	High generalization and robustness; captures neural and behavioral signals	Ding et al. (32); Li et al. (42); Ding et al. (32)
Multimodal (Audio + Video + Text)	F1 = 0.80–0.90	LLM-based Fusion (e.g., AVTF-TBN, MMLA)	High robustness and cross-dataset adaptability	Zhang et al. (20); Sadeghi et al. (35)
EEG + Behavioral (Hybrid)	∼90%	Hybrid CNN-LSTM + Fusion layer	Moderate generalizability; limited by small EEG-behavior datasets	Ay et al. (16); Xia et al. (18)
Speech + Text (Bimodal)	85%–92%	Parallel transformer/Cross-modal Attention	Good generalization; interpretable multimodal embeddings	Li et al. (42); Ding et al. (32)
LLM-driven Multimodal (Audio + Video + Text + Expert Knowledge)	F1 = 0.82–0.90	LLM + Transformer Fusion (Prompt-based)	Highest explainability; adaptable to real-world variability	Sadeghi et al. (35)

**Unimodal vs. multimodal performance:** Unimodal EEG systems remain the strongest single-modality option, with accuracies of 88%–99% in controlled settings (18, 21, 27). However, their performance declines on external datasets due to noise, inter-subject variability, and lack of behavioral or linguistic context. Speech- and text-only systems similarly capture limited aspects of depressive expression. Multimodal models address these gaps by integrating EEG, speech, facial, and linguistic signals and consistently outperform unimodal approaches on datasets such as DAIC–WOZ and E-DAIC. These systems achieve more robust performance (F1 ≈ 0.90) and benefit from attention-based fusion mechanisms that provide interpretable modality weighting.

**Architectural reliability and interpretability:** CNNs and CNN-LSTM hybrids dominate unimodal EEG and speech analysis for their efficiency in modeling spatiotemporal patterns. Transformer-based and cross-modal attention architectures, however, offer superior generalization by dynamically reweighting modality relationships and handling asynchronous inputs. Studies using such architectures (19, 32, 42) report gains in both interpretability and stability across datasets.

**Advantages of large language models (LLMs):** LLM-based systems extend beyond traditional architectures by incorporating contextual reasoning and semantic alignment across modalities, which enhances both prediction quality and interpretability. GPT- and BERT-based multimodal frameworks (35); not only improve the estimation of depression severity but also generate natural-language rationales aligned with clinical symptom descriptions. Studies using LLM-enhanced fusion report greater robustness across datasets and improved handling of heterogeneous inputs compared with conventional deep learning pipelines that depend on handcrafted fusion mechanisms (41, 43). Furthermore, few-shot and zero-shot capabilities reduce reliance on large labeled datasets, providing a scalability advantage in real-world mental health settings where annotated multimodal data remain limited.

**Dataset and demographic variability:** Performance disparities across studies primarily reflect dataset scale and demographic composition. Laboratory datasets (e.g.,SEED-IV) yield inflated accuracies due to controlled conditions (54), whereas community-based datasets such as DAIC–WOZ (55) and E-DAIC produce more conservative but generalizable results (F1 ≈ 0.80–0.90) (56). Demographic underrepresentation remains a persistent limitation.

**Overall synthesis:** Multimodal and LLM-driven architectures currently offer the most reliable and generalizable performance, integrating neurophysiological, behavioral, and linguistic cues within interpretable frameworks. In contrast, unimodal systems—despite strong laboratory results—remain vulnerable to overfitting and limited contextual awareness. The convergence of multimodal fusion and LLM-based reasoning, therefore, represents a key direction for advancing depression detection toward real-world clinical deployment.

### 3.3 Cross-dataset and methodological variability

Although multimodal and transformer-based architectures generally achieve superior performance, substantial variability persists across datasets and fusion strategies due to differences in design, recording conditions, and methodological choices.

**Dataset bias:** Multimodal architectures achieve 90%–95% accuracy on controlled laboratory datasets (e.g., SEED-IV), while yielding F1-scores of 0.80–0.90 on more ecologically valid community datasets such as DAIC-WOZ. reflecting greater linguistic, emotional, and contextual diversity. These disparities illustrate the trade-off between controlled precision and ecological validity, emphasizing the need for training data that capture broad demographic and situational variability.

**Fusion architecture effects:** Fusion design significantly influences robustness and interpretability. Early fusion approaches can perform well on small datasets but suffer from modality imbalance and limited scalability. Late fusion improves modularity yet may overlook fine-grained temporal relationships. Attention-based and transformer architectures offer the most balanced performance by dynamically weighting modality contributions and enabling interpretable cross-modal alignment, both of which are crucial for clinical deployment.

In summary, performance differences across studies largely stem from dataset characteristics and fusion methodology. Future work should prioritize diverse multi-environment datasets and adaptive fusion strategies to support robust and clinically generalizable depression detection systems.

### 3.4 Critical analysis and limitations of current approaches

Despite recent advances, significant methodological and translational limitations persist in automated depression detection research. A central issue is the gap between reported accuracies and real-world generalizability. Many high-performing EEG studies rely on small, homogeneous, single-site datasets, limiting external validity. For example, (21) achieved over 94% accuracy with only 30 subjects, while similarly high results in (18, 27) were based on restricted samples. Comparable concerns appear in (57), where accuracies of 90%–97% were obtained from just 41 participants. These patterns highlight risks of overfitting and dataset-specific learning in controlled environments.

Similar limitations affect multimodal and speech-based systems. Studies such as (32, 58) rely on structured interactions (e.g., VR or DAIC–WOZ), where standardized prompts and recording conditions reduce ecological validity and limit cultural or linguistic generalizability. Reviews by (14, 35) further note the lack of multilingual, multi-accent, and multi-site datasets, which limits cross-lingual robustness.

Evaluation inconsistency also hinders comparability. Metrics such as accuracy, F1-score, MAE, and RMSE capture different aspects of performance, and accuracy can be misleading under class imbalance. Without standardized benchmarks, reproducibility and methodological progress remain difficult to assess.

Fusion-based and multimodal frameworks face additional challenges. Early- and late-fusion approaches often assume synchronous modality alignment, which rarely holds in naturalistic settings. Although transformer and attention mechanisms mitigate misalignment, they depend on large, diverse datasets that remain scarce. This contributes to the gap between laboratory EEG accuracies of 95%–99% (18, 21, 27) and more realistic multimodal results (F1 ≈ 0.78–0.90) (14).

While LLMs offer strong cross-modal alignment and contextual reasoning capabilities, their clinical deployment remains challenged by high computational demands, sensitivity to prompt design, and the risk of generating hallucinated or clinically unreliable outputs. Their general-domain pretraining may not align with clinical semantics, and few studies have compared LLM-generated explanations with clinician judgment, leaving interpretability and reliability concerns unresolved.

Overall, current evidence is constrained by limited dataset diversity, inconsistent evaluation protocols, and insufficient real-world validation. Although deep learning, multimodal fusion, and LLM-based approaches show strong potential, their clinical readiness remains unproven. Progress will require diverse datasets, standardized evaluation frameworks, cross-dataset validation, and clinically grounded interpretability to achieve generalizable and trustworthy systems.

## 4 Discussion

The integration of LLMs in depression detection has consistently outperformed traditional deep learning methods. For instance, studies such as Lau et al. (2023) have shown that LLMs, when fine-tuned, outperform baseline models on the DAIC-WOZ dataset, achieving lower error rates and better handling of diverse multimodal inputs. These findings support the notion that LLMs, due to their ability to generalize better across datasets, offer a promising approach for scalable depression detection in clinical settings. Despite rapid progress in automated depression detection, developing reliable, multimodal, and clinically deployable AI systems remains a significant challenge. Current models often perform well under controlled conditions but struggle to generalize across diverse populations, recording environments, and behavioral contexts. EEG-based systems provide rich neurophysiological information, yet many reported accuracies are inflated due to small, homogeneous samples and laboratory-centered datasets. In contrast, speech-, facial-, and text-based systems operate closer to real-world behavior but are susceptible to demographic bias, cultural variability, and domain shift. These discrepancies highlight the need for more robust methodological foundations and clinically relevant evaluation frameworks.

A primary obstacle is the lack of large, diverse, and standardized multimodal datasets. Existing datasets—whether EEG-based or behavioral—are limited in demographic breadth, linguistic variation, and ecological realism. To improve generalizability, future research must prioritize multi-institutional data collection with harmonized diagnostic labeling and consistent recording protocols. Integrating EEG, speech, facial expressions, language, and other behavioral signals in naturalistic environments would enable models to capture the multifaceted manifestations of depression. Such datasets are essential for meaningful cross-dataset benchmarking and for identifying model biases that remain hidden in single-site studies.

Another critical direction is the development of adaptive and personalized modeling strategies. Depression presents with high inter-individual variability in neural and behavioral patterns, suggesting that one-size-fits-all systems are inherently limited. Meta-learning, domain generalization, and subject-invariant feature representations can help models adjust to personal baselines and evolving symptom trajectories. These approaches also support longitudinal monitoring, relapse prediction, and individualized treatment guidance. Complementing this, real-time and wearable assessment systems—leveraging EEG headsets, smartphone microphones, and ambient sensors—offer feasibility for continuous, privacy-preserving measurement outside laboratory settings, supported by advances in efficient on-device inference.

Large Language Models (LLMs) introduce new opportunities for contextual reasoning, multimodal integration, and explainability. Their ability to align EEG-derived features with linguistic and behavioral cues yields richer, more interpretable representations of depressive states. Techniques such as multimodal instruction tuning, cross-modal embedding alignment, and parameter-efficient fine-tuning (e.g., LoRA, QLoRA, CORAL) further support scalable deployment. However, LLMs also introduce distinct risks: they are computationally intensive, sensitive to prompt design, and susceptible to hallucinations. Few studies have rigorously validated LLM-generated explanations against clinician judgment, leaving open questions about reliability and safety in clinical workflows.

Ethical and fairness considerations must remain central as models transition toward real-world use. Privacy-preserving computation, federated learning, and secure on-device processing are essential for handling sensitive neurobehavioral data. Systems must also be evaluated for demographic fairness across gender, age, culture, and language, and must include interpretability tools that provide transparent, clinically meaningful explanations. Establishing clinician—AI alignment will be crucial for diagnostic trust and regulatory acceptance.

Moving from research prototypes to clinically actionable tools requires rigorous validation across devices, institutions, and patient groups. Standardized evaluation metrics, transparent documentation of preprocessing pipelines, and adherence to regulatory guidelines will be essential. Collaboration among clinicians, AI researchers, industry partners, and regulatory bodies will facilitate safe and reproducible deployment.

In summary, multimodal deep learning, LLM-based contextual reasoning, and large-scale sensing infrastructures offer a promising pathway toward objective and scalable assessment of depression. However, their clinical utility depends on addressing persistent challenges related to dataset diversity, methodological consistency, computational constraints, fairness, and interpretability. By integrating adaptive modeling, ecologically valid data collection, and clinician-aligned explainability, future AI systems can evolve into trustworthy and clinically relevant tools for mental health evaluation. Importantly, ensuring privacy preservation, demographic fairness, transparency, and clinician-aligned oversight will be essential for the safe and responsible translation of these technologies into real-world mental health practice.

## Appendix

**Table A1:** Summary of bias risk and quality assessment for included studies.

Study (Year)	Modality/dataset	Validation method	Key findings	Bias/quality assessment
Acharya et al. (21)	EEG (30 subjects)	10-fold CV	CNN classifier achieved >93% accuracy distinguishing depressed vs. healthy.	High bias—very small sample; possible overfitting.
Mumtaz and Qayyum (22)	EEG (clinical dataset)	Cross-validation	Deep CNN achieved ∼90% accuracy for unipolar depression.	Moderate bias—single-site dataset; limited diversity.
Ay et al. (16)	EEG (controlled lab data)	CNN-LSTM	Combined temporal dependencies; ∼98% accuracy.	High bias—small dataset; overfitting risk.
Kang et al. (23)	EEG	Internal validation	Identified hemispheric EEG asymmetry as biomarker.	Low bias—interpretable; limited population scope.
Seal et al. (24)	EEG (DeprNet)	Within-subject CV	High accuracy (∼97%) using CNN.	Low bias—reproducible, but small dataset.
Uyulan et al. (27)	EEG (n=92)	Subject-independent	eMVAR+deep model; ∼90% accuracy.	Low bias—balanced data; good generalization.
Rafiei et al. (28)	EEG (1-min windows)	Cross-subject	InceptionTime CNN; accuracy >95%.	Moderate bias—short data; limited realism.
Wang et al. (11)	EEG (MODMA)	Augmented CV	Accuracy up to 99.6% (8× augmentation).	Moderate bias—data augmentation may inflate performance.
Xia et al. (18)	EEG (attention-CNN)	Cross-validation	Attention improved interpretability; ∼98% accuracy.	Low bias—interpretable; dataset small.
Sadeghi et al. (35)	Audio+Video+Text (E-DAIC)	Cross-modal fusion	LLM + facial features; MAE = 2.85, RMSE = 4.02.	Moderate bias—single dataset; imbalance present.
Zhang et al. (20)	Audio+Video+Text	Cross-modal validation	AVTF-TBN fusion; F1 = 0.78, Precision = 0.76.	Moderate bias—custom dataset; limited replication.
Lu and Koehn (44)	Text+Audio+Video	Internal CV	LLM2Vec multimodal depression model.	Moderate bias—incomplete dataset description.
Li et al. (42)	Audio+Video+Expert	Parallel Transformer	Expert priors improved PHQ-8 prediction.	Low bias—validated, robust multimodal model.
Ding et al. (32)	Audio+Text (DAIC-WOZ)	Cross-modal attention	F1 = 0.90; strong robustness.	Low bias—public dataset; reproducible.
Yin et al. (19)	Speech (Transformer)	Cross-validation	Combined local spectral+temporal cues.	Moderate bias—small dataset; limited testing.
Zhang et al. (15)	Audio+Video	Cross-dataset	DepITCM fusion; improved accuracy.	Low bias—validated on multiple datasets.
Qin et al. (47)	Text+Interview (LlaMADRS)	Zero-shot prompting	LLM prompting matched clinician ratings.	Low bias—strong benchmarking; robust validation.
Jin et al. (59)	Audio+Facial Video (E-DAIC)	Cross-validation	GCN+LSTM fusion; MAE = 3.51.	Moderate bias—single dataset; good explainability.
Xu et al. (60)	Audio+Video+Text (DAIC-WOZ)	Internal validation	F1=0.85; RMSE = 5.57.	Low bias—solid multimodal performance.
Gan et al. (61)	EEG (frequency fusion)	Cross-validation	EEG spatio-temporal-frequency model.	Moderate bias—limited sample size; preliminary.
