# Correction: Depression detection using deep learning and large language models from multimodalities

**DOI:** 10.3389/fdgth.2026.1827697

**Published:** 2026-03-26

**Authors:** Yasir Hussain, Muhammad Asad Zaheer, Ayaz Muhammad Khan, Aamir Saeed Malik

**Affiliations:** 1 Faculty of Information Technology, Brno University of Technology, Brno, Czechia; 2 Division of Education, University of Education, Lahore, Pakistan

**Keywords:** affective computing, deep learning architectures, EEG-based classification, large language models, multimodal depression detection

The funders Czech Science Foundation, project number 24-10990S and Brno University of Technology, project number FIT-S-26-8984 were erroneously omitted.

The original version of this article has been updated.

